# Understanding and managing atherosclerotic renovascular disease: still a work in progress

**DOI:** 10.12688/f1000research.16369.1

**Published:** 2018-11-29

**Authors:** Alejandro R. Chade

**Affiliations:** 1 Departments of Physiology and Biophysics, Medicine, and Radiology, University of Mississippi Medical Center, 2500 North State Street, Jackson, MS 39216-4505, USA

**Keywords:** renovascular disease, renal angioplasty, microcirculation, angiogenesis, inflammation, mitochondria

## Abstract

Atherosclerotic renovascular disease (ARVD) is an unresolved therapeutic dilemma despite extensive pre-clinical and clinical studies. The pathophysiology of the disease has been widely studied, and many factors that may be involved in progressive renal injury and cardiovascular risk associated with ARVD have been identified. However, therapies and clinical trials have focused largely on attempts to resolve renal artery stenosis without considering the potential need to treat the renal parenchyma beyond the obstruction. The results of these trials show a staggering consistence: although nearly 100% of the patients undergoing renal angioplasty show a resolution of the vascular obstruction, they do not achieve significant improvements in renal function or blood pressure control compared with those patients receiving medical treatment alone. It seems that we may need to take a step back and reconsider the pathophysiology of the disease in order to develop more effective therapeutic strategies. This mini-review discusses potential therapeutic alternatives that focus on the renal parenchyma distal to the vascular obstruction and may provide additional tools to enhance current treatment of ARVD.

Renal artery stenosis is the main cause of chronic renovascular disease (RVD), which in over 90% of cases is due to obstructive atherosclerosis of the main renal artery or primary bifurcations. The buildup of atherosclerotic plaques in the renal arteries is a late reflection of a systemic process that also affects other vascular territories. Indeed, the presence of atherosclerotic RVD (ARVD) may reflect widespread vascular disease, including atherosclerotic disease of the coronary arteries, cerebral blood vessels, and peripheral blood vessels, which together account for the high prevalence of cardiovascular morbidity and vascular-related deaths of patients with ARVD. The widespread nature of atherosclerosis may also be a major contributor to the difficulties in defining a comprehensive therapeutic approach for patients with ARVD. Furthermore, other factors make it challenging to develop a “one size fits all” therapy for ARVD, such as uncertainty regarding the duration of the disease and the likelihood of coexistent comorbidities like pre-existing hypertension, obesity, diabetes, or smoking.

Randomized controlled trials (for example, STAR, DRASTIC, ASTRAL, and CORAL), several smaller studies, and case reports support the notion that interventional strategies that can fully resolve renal artery stenosis may not offer an advantage over medical therapy alone ^1– 4^. These comparative studies show no significant differences between percutaneous transluminal renal angioplasty and stenting (PTRAS) versus medical therapy in outcomes of hypertension, renal function, or cardiovascular risk, consequently failing to justify the inherent risks of a direct intervention (catheter-based with and without stenting or surgical revascularization) on the obstructed renal artery to treat ARVD. Nevertheless, patients who improve their renal function after PTRAS have significantly better survival rates compared with those who do not improve ^5^. Therefore, it might be reasonable to take a step back (or ahead) in ARVD and place current efforts on not only identifying those patients who are most likely to benefit from one strategy or the other and why, but also better understanding the pathophysiology of ARVD. This approach will permit evaluation of the potential for combined strategies that not only address renal artery stenosis, but also protect the renal parenchyma distal to the vascular obstruction.

## The bumps in the road

ARVD is a complex disease. An inherent challenge in the diagnosis of ARVD (via ultrasound of the renal arteries, magnetic resonance angiography, or contrast-enhanced angiogram) ^6^ is the frequent inaccuracy of the angiographic determination of the severity of the stenosis, which may contribute to a delayed diagnosis and missed opportunities for interventions with predictable outcomes ^4^. Another challenge is that no specific clinical characteristic in patients with ARVD can reliably predict the renal functional outcome after treatment ^7^. Furthermore, for several years, all the attention was on resolving the renal vascular obstruction. It is undeniable that a severe, hemodynamically significant stenosis (defined as a reduction of at least 70% of the arterial lumen or at least 50% of the area) is a prominent insult for the development of renal ischemia and hypertension, as has been shown in elegant studies using translational animal models of ARVD as well as in the clinical setting. Nevertheless, the results of the two largest randomized controlled trials—ASTRAL and CORAL—that performed a comparative evaluation of the therapeutic efficacy of PTRAS versus full medical therapy resulted in the boldest testimony *against* catheter-based strategies for ARVD ^1, 2, 8^: there seems to be no significant advantage of PTRAS over medical therapy.

However, open questions remain. Recent scrutiny of these studies highlighted important flaws in the design that may have played a role in some of the clear but often misleading results and subsequent conclusions. In-depth discussion of their design strengths and weaknesses is beyond the scope of this article, and the reader may consult recently published literature ^4, 9^. A major criticism of these trials includes the fact that almost 50% of the recruited patients showed renal artery stenosis of less than 70% and normal renal function or subclinical (stage I or II) renal dysfunction ^3^. Thus, clinical characteristics (underlying pathophysiology) of almost half of the patients may have played a role in the indifferent results of the trials. Furthermore, evidence obtained by subanalysis of these trials supports the notion that renal artery stenosis of at least 80% (associated or not with severe uncontrolled hypertension), patients with a rapid deterioration of renal function, and patients presenting with flash pulmonary edema distinctly benefited from PTRAS ^3, 10, 11^. The selective but limited beneficial effects of PTRAS for ARVD highlight the complexity of the disease and suggest that factors prior to and beyond the vascular obstruction may likely be determinants in the outcome. These results also emphasize that vascular obstruction is an important component but may not be the only abnormality that should be treated. Finally, the provocative findings of these subanalyses support the notion that therapeutic approaches should be carefully individualized and tailored for different patients.

## Trying to fix some of the bumps

Aggressive control of cardiovascular risk factors often associated with ARVD and chronic kidney disease (CKD), such as hypertension, dyslipidemia, metabolic abnormalities, and lifestyle modifications, is part of the current therapeutic arsenal for these patients and will not be discussed in this mini-review. Instead, we will focus on recent strategies aimed to protect the renal parenchyma with translational potential to serve as possible additions to current treatments or as stand-alone therapies.

The degree of functional and structural damage of the renal parenchyma distal to the stenosis may be a critical but often unattended assessment for the selection of therapeutic strategies in ARVD. The kidney is a complex organ in which the vasculature plays multiple roles by providing nutrition of renal tissue, filtration of blood, and removal of systemic waste. Studies from pre-clinical and clinical settings showed that renal microvascular rarefaction, both functional and structural, is a universal pathological feature of CKD that may start early in prominent cardiovascular and renal risk factors like hypertension, diabetes, or obesity and may often predict decline in renal function ^12– 18^. Pre-clinical studies also show that ARVD induces a significant and often progressive remodeling and loss of the microvasculature distal to the stenosis, paired with blunted renal mechanisms of microvascular repair and angiogenesis ^19, 20^. The progressive microvascular injury is the result of combined insults such as renal ischemia, systemic and renal inflammation, and consequent pro-fibrotic and pro-apoptotic activity that develops in the kidney, often independently of the severity of renal artery stenosis but exacerbated when several cardiovascular risk factors coexist ^21, 22^. Moreover, experimental studies showed that PTRAS does not fully recover renal function (despite fully resolving the stenosis) and that microvascular rarefaction and renal fibrosis persist ^23, 24^. Furthermore, renal microvascular endothelial cells display poor tolerance to injury paired with limited regenerative capacity, which suggests a distinct environment for early development of renal damage ^25^. All together, these data suggest that the selection of patients suitable for catheter-based interventions should be driven by the anatomic grading of the stenosis and functional assessment of the renal microvasculature since, like the coronary circulation, functional measurements may help to better predict the therapeutic efficacy of PTRAS in ARVD ^26^. In other words, restoration of blood flow into the kidneys may be futile if a severely or irreversibly dysfunctional renal parenchyma is the recipient, and it should not be neglected.

## Emerging strategies to protect the renal parenchyma: a chance for atherosclerotic renovascular disease?

Recent studies support cell-based therapies for the kidney as a feasible option for renoprotection. Intra-renal administration of autologous endothelial or mesenchymal progenitor cells induced significant protection of the renal microvascular architecture (cortex and medulla) that seems to drive functional recovery as well ^27– 30^. The effects of cell-based therapies may not be limited to neovascularization or microvascular repair or both. These are pluripotential cells that are mobilized in response to insults in order to build (or restore) pivotal endogenous mechanisms of tissue repair. Indeed, given the diverse cargo of endothelial and mesenchymal cells (for example, extracellular vesicles ^31– 33^), it is possible that these cells participate in modulating multiple pathophysiological pathways—such as inflammation, apoptosis, and fibrosis—that may contribute to a steady renal improvement. Thus, this approach represents an excellent opportunity for subsequent exploration of clinical applications in ARVD. Pre-clinical evidence supports their potential, and ongoing clinical trials (for example, ClinicalTrials.gov Identifier: NCT02266394) will determine the safety and efficacy of this strategy for future clinical use.

Targeted strategies for the renal microvasculature are also being tested. Recent experimental studies using intra-stenotic kidney administration of hepatocyte growth factor ^34^ or vascular endothelial growth factor (VEGF) ^35^ in experimental swine ARVD resulted in substantial microvascular repair and neovascularization that correlated with recovery of renal blood flow and filtration function, suggesting that new and repaired microvessels were functional. Furthermore, co-adjuvant VEGF therapy improved the outcomes of PTRAS, emphasizing the notion that the status of the renal parenchyma may play a pivotal role in renal recovery ^23^. Therapeutic angiogenesis for the kidney is a strategy with high potential and is currently under development and refinement. Indeed, ongoing work using drug-delivery technologies to enhance kidney targeting of VEGF in ARVD ^36– 38^ and CKD (author’s unpublished work in progress) may boost the translational potential of these strategies.

Another emerging target being considered for renal therapies is the mitochondria. Several forms of acute and chronic renal disease have been associated with changes in mitochondrial homeostasis, formation (biogenesis), morphology, and degradation (mitophagy), and studies show that this plethora of abnormalities may lead to reduced ATP generation, altered calcium signaling, oxidative stress, and cell death ^39^. Although evidence comes mainly from pre-clinical settings (and a few ongoing clinical trials), mitochondrial targeting (via specific inhibition of mitochondrial permeability transition pore opening) shows potential as a feasible strategy to preserve mitochondrial structure and function, restore biogenesis, and ameliorate kidney injury in experimental ARVD (with and without PTRAS ^40, 41^), hypertension ^42^, and metabolic syndrome-induced renal damage ^43^. Thus, therapeutic targeting of the mitochondria, being key organelles in cell metabolism and function, could serve as a strategy that induces recovery in the vascular, tubular, and glomerular compartments and has the potential for applications to renal disease of different etiologies and stages.

The development and progression of systemic and renal inflammation are major pathological mechanisms in chronic renal disease of any etiology. Elevation in biomarkers suggestive of inflammation (for example, C-reactive protein, neutrophil gelatinase-associated lipocalin [NGAL], or tumor necrosis factor receptor ^44^), inflammatory cytokines (for example, interleukins and tumor necrosis factor-alpha ^45^), and infiltration of inflammatory cells in the renal parenchyma (for example, neutrophils, lymphocytes, and macrophages ^46, 47^) may be observed early, during subclinical stages of CKD, and increase in parallel as CKD advances ^44^. Therefore, biomarkers may be able to evaluate the inflammatory state in kidney disease and help to predict cardiovascular risk. Progressive systemic and renal inflammations are pathological footprints of CKD ^48^ and major determinants for the progression to end-stage renal disease and higher cardiovascular and all-cause mortality ^49– 51^. The major etiology of ARVD is atherosclerosis, which is characterized by a chronic low-grade inflammatory state and can instigate inflammatory infiltrates in the kidney ^49, 52^. Previous studies aimed to offset inflammation in CKD from diverse etiologies by blocking cytokines that activate, recruit, and are produced by inflammatory cells (for example, macrophages) or by stimulating their depletion ^50, 53^. Targeting inflammation has not been tested in ARVD and offers an open field as a novel intervention that warrants further studies.

Chronic inflammation may precede and favor the development of renal fibrosis, a common final pathway of chronic renal diseases irrespective of the etiology. Although fibrosis may be absent, minimal, or at a relatively early stage in ARVD (compared to CKD), the stenotic kidney offers a favorable milieu in which the convergence of insults such as amplified activation of the renin–angiotensin system, oxidative stress, ischemia, inflammation, and microvascular rarefaction may aggravate kidney damage and favor the development of renal fibrosis ^46, 54^. Given that renal fibrosis likely is an irreversible stage of loss of functional renal tissue that affects all renal compartments, strategies to prevent or stop its development in ARVD could have a significant impact.

Efforts to develop potential anti-fibrotic strategies for the kidney have been made, with a major focus on anti-transforming growth factor-beta (anti-TGF-β) strategies (for example, inhibition of synthesis, antibodies ^55, 56^) but also on other factors such as connective tissue growth factor inhibitors, epidermal growth factor inhibitors, or bone morphogenetic factor agonists ^57^. However, although promising results are observed in pre-clinical studies, conclusive evidence regarding the usefulness of many of the targeted anti-fibrotic strategies is limited, and the understanding of the long-term efficacy and safety of these agents to treat renal fibrosis warrants larger studies. Currently, there are still no clinical therapies in use that specifically target renal cells or kidney fibrosis. Extensive discussion of specific anti-fibrotic strategies and targets is beyond the scope of this brief review, and the reader is encouraged to consult published work ^57, 58^.

## Conclusions and perspectives

The kidney is a highly complex organ and therefore therapies are often difficult to fully define. Several tools are available to induce protection of the renal compartments and are part of the standard pharmacotherapy for patients with renal disease. These therapies include inhibitors of the renin–angiotensin system, antioxidants, and lipid-lowering and glucose-control drugs to counteract the deleterious effects on renal function of pathologic conditions that may initiate, accompany, and contribute to the progression of acute and chronic renal disease. Unfortunately, the progressive nature of chronic renal diseases imposes a significant barrier to comprehensive therapies, which should usually be adapted to the patient.

**Figure 1.**
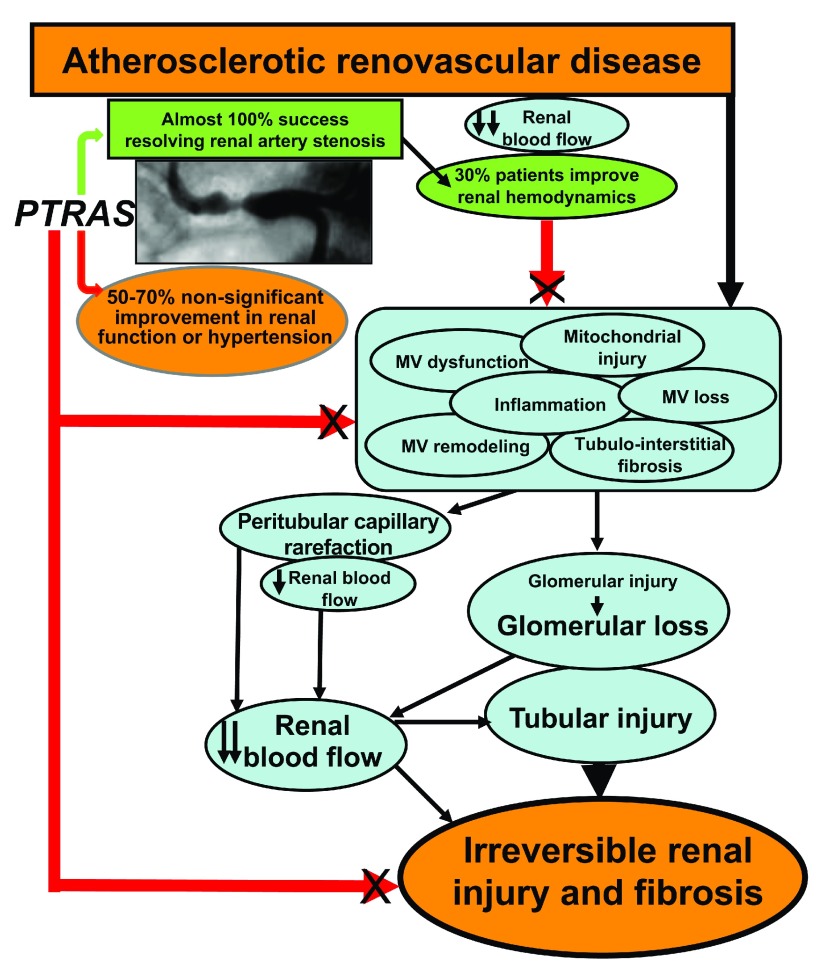
Schematic overview of the pathophysiology of atherosclerotic renovascular disease and hypothesis of the outcomes of percutaneous transluminal renal angioplasty and stenting (PTRAS). Despite the high success of PTRAS in resolving renal artery stenosis (green arrow and green box), a limited recovery of blood pressure control and renal function is consistently observed (green and orange ovals). This disparity may be due to unresolved progressive pathophysiological mechanisms in the kidney (light blue ovals), distal to the stenosis, that are not addressed by the sole restoration of blood flow by PTRAS (red arrows, black X) and may continue to deteriorate the renal parenchyma. MV, microvascular.

The therapeutic dilemma of ARVD is still a work in progress in which the emphasis placed on resolving renal artery stenosis has overlooked the renal parenchyma, distal to the obstruction ( Figure 1). Renoprotective strategies that combine resolution of the stenosis with novel co-adjuvant interventions to preserve, protect, and improve renal outcomes and reduce cardiovascular risk in patients with ARVD should be on the horizon. Efforts should focus on the design and testing of novel comprehensive strategies to protect the renal parenchyma in ARVD, likely in addition to PTRAS. Some novel therapies with translational potential discussed in this brief review are showing promising results in relevant models of ARVD and may change the current paradigm. These are feasible therapeutic alternatives that should be considered and could be tailored to the patient in a timely fashion.

## Abbreviations

ARVD, atherosclerotic renovascular disease; CKD, chronic kidney disease; PTRAS, percutaneous transluminal renal angioplasty and stenting; RVD, renovascular disease; VEGF, vascular endothelial growth factor

## References

[1] Cooper CJ Murphy TP Cutlip DE: Stenting and medical therapy for atherosclerotic renal-artery stenosis. *N Engl J Med.* 2014;370(1):13–22. 10.1056/NEJMoa1310753 24245566 PMC4815927

[2] ASTRAL Investigators, Wheatley K Ives N: Revascularization versus medical therapy for renal-artery stenosis. *N Engl J Med.* 2009;361(20):1953–62. 10.1056/NEJMoa0905368 19907042

[3] Mohan IV Bourke V: The management of renal artery stenosis: an alternative interpretation of ASTRAL and CORAL. *Eur J Vasc Endovasc Surg.* 2015;49(4):465–73. 10.1016/j.ejvs.2014.12.026 25725508

[4] White CJ: Optimizing outcomes for renal artery intervention. *Circ Cardiovasc Interv.* 2010;3(2):184–92. 10.1161/CIRCINTERVENTIONS.109.910208 20407115

[5] Tafur JD White CJ: Renal Artery Stenosis: When to Revascularize in 2017. *Curr Probl Cardiol.* 2017;42(4):110–35. 10.1016/j.cpcardiol.2017.01.004 28325353

[6] Salifu MO Haria DM Badero O: Challenges in the diagnosis and management of renal artery stenosis. *Curr Hypertens Rep.* 2005;7(3):219–27. 10.1007/s11906-005-0014-3 15913498

[7] Ronden RA Houben AJ Kessels AG: Predictors of clinical outcome after stent placement in atherosclerotic renal artery stenosis: a systematic review and meta-analysis of prospective studies. *J Hypertens.* 2010;28(12):2370–7. 20811293 10.1097/HJH.0b013e32833ec392

[8] Cheung CM Chrysochou C Kalra PA: The management of renovascular disease: ASTRAL and beyond. *Curr Opin Nephrol Hypertens.* 2011;20(1):89–94. 10.1097/MNH.0b013e328340ffe5 21045682

[9] Mousa AY AbuRahma AF Bozzay J: Update on intervention versus medical therapy for atherosclerotic renal artery stenosis. *J Vasc Surg.* 2015;61(6):1613–23. 10.1016/j.jvs.2014.09.072 26004332 PMC4663460

[10] Alderson HV Ritchie JP Kalra PA: Revascularization as a treatment to improve renal function. *Int J Nephrol Renovasc Dis.* 2014;7:89–99. 10.2147/IJNRD.S35633 24600242 PMC3933706

[11] Ritchie J Green D Chrysochou C: High-risk clinical presentations in atherosclerotic renovascular disease: prognosis and response to renal artery revascularization. *Am J Kidney Dis.* 2014;63(2):186–97. 10.1053/j.ajkd.2013.07.020 24074824

[12] Futrakul N Futrakul P: Renal microvascular disease predicts renal function in diabetes. *Ren Fail.* 2012;34(1):126–9. 10.3109/0886022X.2011.623490 22010784

[13] Grassi G Schiffrin EL: Media-to-lumen ratio as predictor of renal abnormalities in hypertension: new findings, new questions. *J Hypertens.* 2010;28(9):1811–3. 10.1097/HJH.0b013e32833d7fed 20699714

[14] Maric-Bilkan C Flynn ER Chade AR: Microvascular disease precedes the decline in renal function in the streptozotocin-induced diabetic rat. *Am J Physiol Renal Physiol.* 2012;302(3):F308–15. 10.1152/ajprenal.00421.2011 22031855 PMC3287355

[15] Schiffrin EL: Vascular remodeling in hypertension: mechanisms and treatment. *Hypertension.* 2012;59(2):367–74. 10.1161/HYPERTENSIONAHA.111.187021 22203749

[16] Ehling J Bábíčková J Gremse F: Quantitative Micro-Computed Tomography Imaging of Vascular Dysfunction in Progressive Kidney Diseases. *J Am Soc Nephrol.* 2016;27(2):520–32. 10.1681/ASN.2015020204 26195818 PMC4724942

[17] Mack M Yanagita M: Origin of myofibroblasts and cellular events triggering fibrosis. *Kidney Int.* 2015;87(2):297–307. 10.1038/ki.2014.287 25162398

[18] Xavier S Vasko R Matsumoto K: Curtailing endothelial TGF- *β* signaling is sufficient to reduce endothelial-mesenchymal transition and fibrosis in CKD. *J Am Soc Nephrol.* 2015;26(4):817–29. 10.1681/ASN.2013101137 25535303 PMC4378095

[19] Chade AR: Renal vascular structure and rarefaction. *Compr Physiol.* 2013;3(2):817–31. 10.1002/cphy.c120012 23720331 PMC3936257

[20] Chade AR: Small Vessels, Big Role: Renal Microcirculation and Progression of Renal Injury. *Hypertension.* 2017;69(4):551–63. 10.1161/HYPERTENSIONAHA.116.08319 28193706 PMC5344725

[21] Chade AR Rodriguez-Porcel M Grande JP: Distinct renal injury in early atherosclerosis and renovascular disease. *Circulation.* 2002;106(9):1165–71. 10.1161/01.CIR.0000027105.02327.48 12196346

[22] Chade AR Rodriguez-Porcel M Grande JP: Mechanisms of renal structural alterations in combined hypercholesterolemia and renal artery stenosis. *Arterioscler Thromb Vasc Biol.* 2003;23(7):1295–301. 10.1161/01.ATV.0000077477.40824.52 12750121

[23] Chade AR Kelsen S: Renal microvascular disease determines the responses to revascularization in experimental renovascular disease. *Circ Cardiovasc Interv.* 2010;3(4):376–83. 10.1161/CIRCINTERVENTIONS.110.951277 20587789 PMC3032938

[24] Chade AR Tullos N Stewart NJ: Endothelin-a receptor antagonism after renal angioplasty enhances renal recovery in renovascular disease. *J Am Soc Nephrol.* 2015;26(5):1071–80. 10.1681/ASN.2014040323 25377076 PMC4413765

[25] Basile DP Zeng P Friedrich JL: Low proliferative potential and impaired angiogenesis of cultured rat kidney endothelial cells. *Microcirculation.* 2012;19(7):598–609. 10.1111/j.1549-8719.2012.00193.x 22612333 PMC3458172

[26] van Brussel PM van de Hoef TP de Winter RJ: Hemodynamic Measurements for the Selection of Patients With Renal Artery Stenosis: A Systematic Review. *JACC Cardiovasc Interv.* 2017;10(10):973–85. 10.1016/j.jcin.2017.02.046 28521931

[27] Chade AR Zhu X Lavi R: Endothelial progenitor cells restore renal function in chronic experimental renovascular disease. *Circulation.* 2009;119(4):547–57. 10.1161/CIRCULATIONAHA.108.788653 19153272 PMC2758066

[28] Chade AR Zhu XY Krier JD: Endothelial progenitor cells homing and renal repair in experimental renovascular disease. *Stem Cells.* 2010;28(6):1039–47. 10.1002/stem.426 20506499 PMC2958683

[29] Ebrahimi B Eirin A Li Z: Mesenchymal stem cells improve medullary inflammation and fibrosis after revascularization of swine atherosclerotic renal artery stenosis. *PLoS One.* 2013;8(7):e67474. 10.1371/journal.pone.0067474 23844014 PMC3701050

[30] Eirin A Zhu XY Krier JD: Adipose tissue-derived mesenchymal stem cells improve revascularization outcomes to restore renal function in swine atherosclerotic renal artery stenosis. *Stem Cells.* 2012;30(5):1030–41. 10.1002/stem.1047 22290832 PMC3694782

[31] Eirin A Zhu XY Jonnada S: Mesenchymal Stem Cell-Derived Extracellular Vesicles Improve the Renal Microvasculature in Metabolic Renovascular Disease in Swine. *Cell Transplant.* 2018;27(7):1080–95. 10.1177/0963689718780942 29954220 PMC6158551

[32] Eirin A Zhu XY Puranik AS: Mesenchymal stem cell-derived extracellular vesicles attenuate kidney inflammation. *Kidney Int.* 2017;92(1):114–24. 10.1016/j.kint.2016.12.023 28242034 PMC5483390

[33] Eirin A Zhu XY Puranik AS: Integrated transcriptomic and proteomic analysis of the molecular cargo of extracellular vesicles derived from porcine adipose tissue-derived mesenchymal stem cells. *PLoS One.* 2017;12(3):e0174303. 10.1371/journal.pone.0174303 28333993 PMC5363917

[34] Stewart N Chade AR: Renoprotective effects of hepatocyte growth factor in the stenotic kidney. *Am J Physiol Renal Physiol.* 2013;304(6):F625–33. 10.1152/ajprenal.00504.2012 23269649 PMC3602702

[35] Chade AR Kelsen S: Reversal of renal dysfunction by targeted administration of VEGF into the stenotic kidney: a novel potential therapeutic approach. *Am J Physiol Renal Physiol.* 2012;302(10):F1342–50. 10.1152/ajprenal.00674.2011 22357917 PMC3362061

[36] Bidwell GL 3rd Mahdi F Shao Q: A kidney-selective biopolymer for targeted drug delivery. *Am J Physiol Renal Physiol.* 2017;312(1):F54–F64. 10.1152/ajprenal.00143.2016 27784692 PMC5283886

[37] Chade AR Tullos NA Harvey TW: Renal Therapeutic Angiogenesis Using a Bioengineered Polymer-Stabilized Vascular Endothelial Growth Factor Construct. *J Am Soc Nephrol.* 2016;27(6):1741–52. 10.1681/ASN.2015040346 26541349 PMC4884109

[38] Chade AR Williams ML Guise E: Systemic biopolymer-delivered vascular endothelial growth factor promotes therapeutic angiogenesis in experimental renovascular disease. *Kidney Int.* 2018;93(4):842–54. 10.1016/j.kint.2017.09.029 29273331 PMC5866753

[39] Eirin A Lerman A Lerman LO: Mitochondrial injury and dysfunction in hypertension-induced cardiac damage. *Eur Heart J.* 2014;35(46):3258–66. 10.1093/eurheartj/ehu436 25385092 PMC4258226

[40] Eirin A Ebrahimi B Zhang X: Mitochondrial protection restores renal function in swine atherosclerotic renovascular disease. *Cardiovasc Res.* 2014;103(4):461–72. 10.1093/cvr/cvu157 24947415 PMC4155472

[41] Eirin A Li Z Zhang X: A mitochondrial permeability transition pore inhibitor improves renal outcomes after revascularization in experimental atherosclerotic renal artery stenosis. *Hypertension.* 2012;60(5):1242–9. 10.1161/HYPERTENSIONAHA.112.199919 23045468

[42] Eirin A Lerman A Lerman LO: Mitochondria: a pathogenic paradigm in hypertensive renal disease. *Hypertension.* 2015;65(2):264–70. 10.1161/HYPERTENSIONAHA.114.04598 25403611 PMC4289015

[43] Eirin A Hedayat AF Ferguson CM: Mitoprotection preserves the renal vasculature in porcine metabolic syndrome. *Exp Physiol.* 2018;103(7):1020–9. 10.1113/EP086988 29714040 PMC6026037

[44] Sharain K Hoppensteadt D Bansal V: Progressive increase of inflammatory biomarkers in chronic kidney disease and end-stage renal disease. *Clin Appl Thromb Hemost.* 2013;19(3):303–8. 10.1177/1076029612454935 22865783

[45] Spoto B Leonardis D Parlongo RM: Plasma cytokines, glomerular filtration rate and adipose tissue cytokines gene expression in chronic kidney disease (CKD) patients. *Nutr Metab Cardiovasc Dis.* 2012;22(11):981–8. 10.1016/j.numecd.2011.01.005 21906921

[46] Eddy AA: Overview of the cellular and molecular basis of kidney fibrosis. *Kidney Int Suppl (2011).* 2014;4(1):2–8. 10.1038/kisup.2014.2 25401038 PMC4220516

[47] Rabb H Griffin MD McKay DB: Inflammation in AKI: Current Understanding, Key Questions, and Knowledge Gaps. *J Am Soc Nephrol.* 2016;27(2):371–9. 10.1681/ASN.2015030261 26561643 PMC4731128

[48] Stenvinkel P Alvestrand A: Inflammation in end-stage renal disease: sources, consequences, and therapy. *Semin Dial.* 2002;15(5):329–37. 10.1046/j.1525-139X.2002.00083.x 12358637

[49] Akchurin OM Kaskel F: Update on inflammation in chronic kidney disease. *Blood Purif.* 2015;39(1–3):84–92. 10.1159/000368940 25662331

[50] Rodríguez-Iturbe B Pons H Herrera-Acosta J: Role of immunocompetent cells in nonimmune renal diseases. *Kidney Int.* 2001;59(5):1626–40. 10.1046/j.1523-1755.2001.0590051626.x 11318933

[51] Sean Eardley K Cockwell P: Macrophages and progressive tubulointerstitial disease. *Kidney Int.* 2005;68(2):437–55. 10.1111/j.1523-1755.2005.00422.x 16014021

[52] Chade AR Rodriguez-Porcel M Herrmann J: Antioxidant intervention blunts renal injury in experimental renovascular disease. *J Am Soc Nephrol.* 2004;15(4):958–66. 10.1097/01.ASN.0000117774.83396.E9 15034098

[53] Huang L Wang A Hao Y: Macrophage Depletion Lowered Blood Pressure and Attenuated Hypertensive Renal Injury and Fibrosis. *Front Physiol.* 2018;9:473. 10.3389/fphys.2018.00473 29867533 PMC5949360

[54] Lerman LO Textor SC Grande JP: Mechanisms of tissue injury in renal artery stenosis: ischemia and beyond. *Prog Cardiovasc Dis.* 2009;52(3):196–203. 10.1016/j.pcad.2009.09.002 19917330 PMC2800096

[55] Cho ME Kopp JB: Pirfenidone: an anti-fibrotic therapy for progressive kidney disease. *Expert Opin Investig Drugs.* 2010;19(2):275–83. 10.1517/13543780903501539 20050822 PMC3058482

[56] Takakuta K Fujimori A Chikanishi T: Renoprotective properties of pirfenidone in subtotally nephrectomized rats. *Eur J Pharmacol.* 2010;629(1–3):118–24. 10.1016/j.ejphar.2009.12.011 20006961

[57] Klinkhammer BM Goldschmeding R Floege J: Treatment of Renal Fibrosis-Turning Challenges into Opportunities. *Adv Chronic Kidney Dis.* 2017;24(2):117–29. 10.1053/j.ackd.2016.11.002 28284377

[58] Lee SY Kim SI Choi ME: Therapeutic targets for treating fibrotic kidney diseases. *Transl Res.* 2015;165(4):512–30. 10.1016/j.trsl.2014.07.010 25176603 PMC4326607

